# Atypical Presentation of Probable Sporadic Creutzfeldt-Jakob Disease: A Patient Without Mental Deterioration

**DOI:** 10.7759/cureus.64814

**Published:** 2024-07-18

**Authors:** Nuzhat Nisa, Novera Inam, Christopher Stewart, Suporn Sukpraprut-Braaten

**Affiliations:** 1 Family Medicine, Reid Health, Richmond, USA; 2 Medicine, Kansas City University of Medicine and Biosciences, Kansas City, USA; 3 Graduate Medical Education, Kansas City University of Medicine and Biosciences, Kansas City, USA

**Keywords:** 14-3-3 protein, atypical presentation of creutzfeldt jakob disease, creutzfeldt jakob disease imaging, neurology and critical care, creutzfeldt jakob disease

## Abstract

Creutzfeldt-Jakob Disease (CJD) is a prion disease that leads to rapid mental deterioration and is always fatal. Prions are glycoproteins found in the brain. While their function is not completely understood, irregular folding of these proteins leads to prion disorders and neurodegenerative disease. CJD is extremely rare (1-2 cases per million people). A 68-year-old woman presented to the family medicine clinic with symptoms of weakness, paresthesia, and foot drop. Some weeks later she presented at the emergency department with left ankle and foot pain. All symptoms were on the left side of the body. An initial workup with labs was performed which all returned normal. Subsequently, a cerebrospinal fluid (CSF) panel was run and findings included elevated neuron-specific enolase and 14-3-3 gamma indicating a neurodegenerative disease. Further, an indeterminate real-time quaking-induced conversion (RT-QuIC) led to our diagnosis of a probable sporadic CJD. The patient was treated for symptoms and died four months following the initial presentation. Typically CJD presents with similar physical symptoms such as myoclonus. CJD is typically accompanied by severe mental deterioration including depression, memory loss, and dementia. CJD presentation without mental deterioration has only been reported in two other cases. Presenting here is a unique presentation of probable CJD that involved all the physical symptoms, including death, but the mental deterioration was absent. Clinicians should be aware of CJD and that presentation is not always standard.

## Introduction

Creutzfeldt-Jakob disease (CJD) was first encountered in the early 1920s by German neuropathologists Alfons Maria Jakob and Hans Gerhardt Creutzfeldt, who saw patients with dementia, neurodegeneration, and spasticity [1]. Typical clinical presentations of CJD include severe mental deterioration and dementia accompanied by myoclonus or involuntary muscle movement [2]. Additional symptoms commonly seen are slurred speech, numbness, hallucinations, depression, anxiety, and difficulty speaking [3]. Currently, there are three categories of CJD- sporadic, familial, or iatrogenic, with more than 80% of the cases reported as sporadic [4]. The neurodegenerative symptoms observed in CJD are caused by abnormal forms of prion proteins [4]. The functional role of prions is not entirely understood; however, abnormal prions spread, aggregate, and lead to disease [4]. One of the common suspicions for CJD is a sudden onset of dementia, clonus, and other motor symptoms [4]. However, the presentation can be variable [4]. There is no current treatment for CJD and 70% of cases result in death within one year of diagnosis [2].

## Case presentation

A 68-year-old female presented at the Family Medicine clinic with left-sided weakness, paresthesia, and foot drop. She later reported to the emergency department with left ankle and foot pain. She also reported myoclonus and urinary incontinence that had progressively worsened over a two-month period. The patient had no issues using the right side of her body. She had no slurred speech, facial droop, or cognitive deficit. The patient also had no significant behavioral or mental changes, although no cognitive assessment was done. She had no previous surgeries, no medications, and no remarkable medical history. The patient reported paternal cerebellar ataxia.

An X-ray of the left foot, ankle, and spine revealed no abnormalities. An electromyography (EMG) was next done which also came back with no abnormalities. Next magnetic resonance imaging (MRI) was done of the lumbar and spinal cord which showed multilevel degenerative changes and central canal stenosis at C4-5 and C5-6 with some indentation but nothing that would explain the patient’s symptoms. Next labs were taken to develop a diagnosis (Table 1), including a meningitis panel which came back negative. An EEG was also ordered which returned with abnormal findings.

**Table 1:** Lab values from Creutzfeldt Jakob disease (CJD) workup *: abnormal lab value used in CJD diagnosis TSH: thyroid stimulating hormone; CSF: cerebrospinal fluid; WNL: within normal physiologic limits; dsDNA: double-stranded deoxyribonucleic acid; RT-QuIC: real-time quaking-induced conversion

Test	Patient	Normal
Meningitis panel	Negative	Negative
Ammonia, folate, TSH	WNL	WNL
Oligoclonal bands (CSF)	Negative	Negative
Varicella/West Nile virus (CSF)	Negative	Negative
Thyroid peroxidase A, antinuclear antibody, dsDNA Ab, rheumatoid factor	WNL	WNL
Autoimmune panel	Negative	Negative
Vitamin B12	>7500 pg/mL	180-914 pg/mL
Neuron specific enolase* (CSF)	27.1 ng/mL	21.5 ng/mL
Myelin basic protein (CSF)	2.03 ng/mL	0-5 ng/mL
RT-QuIC (CSF)*	indeterminate	negative
T-tau protein (CSF)	898 pg/mL	0-1149 pg/mL
14-3-3 gamma (CSF)*	5638 AU/mL	<30-1999 AU/mL

**Figure 1:**
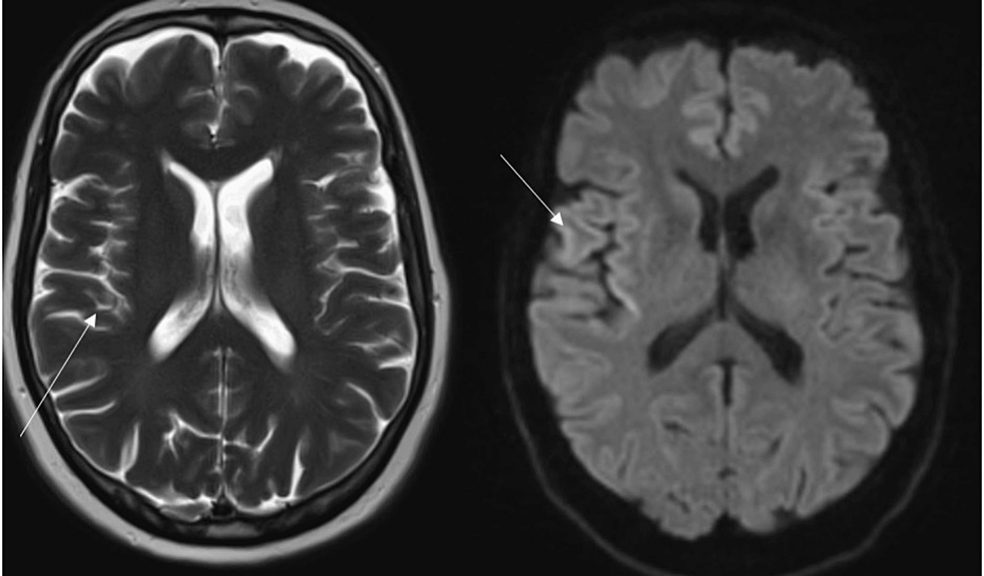
MRI showing hyper-intensity along the frontal and temporo-parieto-occipital region. White arrows indicate hyper-intensity

Significant findings from the cerebrospinal fluid (CSF) sample include elevated levels of neuron-specific enolase and 14-3-3 Gamma (Table 1). These are both nonspecific indicators of a neurodegenerative disease. Based on these findings an MRI was ordered (Figure 1). Cerebral MRI revealed increased diffusion and fluid-attenuated inversion recovery (FLAIR) at the cortex of the posterior right parietal lobe along with hyperintensity/cortical ribboning at the frontal and temporo-parietal-occipital region (Figure 1). Differential diagnoses from the MRI include the non-specific findings of encephalitis, hepatic encephalopathy, hypoglycemic encephalopathy, and chronic white-matter changes. A 1.4 cm benign extra-axial structure adjacent to the right frontal lobe was also found. We are doubtful this structure has any clinical significance. Based on these findings a cerebrospinal fluid real-time quaking-induced conversion (RT-QuIC) test was ordered. RT-QuIC is a highly specific test for sporadic CJD. The test came back indeterminate.

Criteria for a sporadic CJD diagnosis are found in Table 2. One symptom from each category is needed for a CJD diagnosis. Based on the indeterminate RT-QuIC, myoclonus, pyramidal dysfunction, hyperintensity in the temporo-parietal-occipital region, positive 14-3-3 gamma assay, and FLAIR signal in cerebral MRI, the patient was diagnosed with probable sporadic Creutzfeldt Jakob’s Disease (sCJD). We concluded that it was sporadic because the patient was not exposed to any potential agents, and there was no genetic or family history. No genetic testing was performed due to a lack of funding.

**Table 2:** UpToDate criteria for diagnosing CJD versus patient RT-QuIC: real-time quaking-induced conversion; EEG: electroencephalogram; PSWC: period sharp wave complexes; CSF: cerebrospinal fluid; MRI: magnetic resonance imaging; DWI: diffusion-weighted imaging; FLAIR: fluid-attenuated inversion recovery; CJD: Creutzfeldt Jakob disease Reference: [5]

Diagnostic criteria for Creutzfeldt Jakob disease	
Criteria	Patient
One of the following: neuropsychiatric disorder with a positive real-time quaking-induced conversion (RT-QuIC) test. Progressive dementia.	Patient had an indeterminate RT-QuIC test and no progressive dementia.
Two of the following: myoclonus visual or cerebellar disturbance pyramidal or extrapyramidal dysfunction akinetic mutism.	Patient presented with myoclonus and pyramidal dysfunction.
One of the following: Atypical EEG (e.g., periodic sharp wave complexes (PSWC)) during an illness of any duration Positive 14-3-3 cerebrospinal fluid (CSF) assay with a clinical duration to death less than two years MRI showing hyperintensity in caudate nucleus/putamen and/or in at least two cortical regions (temporal, parietal, and occipital) on diffusion-weighted imaging (DWI) or fluid-attenuated inversion recovery (FLAIR).	MRI showed hyperintensity along the frontal and temporo-parieto-occipital region along with FLAIR in the cerebral cortex. An EEG was also done which was normal. Patient also had elevated 14-3-3 CSF levels and died four months following labs.
Routine investigations should not suggest an alternative diagnosis.	Patient was treated symptomatically and died four months later.

The patient was treated symptomatically for myoclonus using anti-seizure medications. She was also referred to physical therapy due to muscle weakness and quickly became wheelchair-bound. Muscle weakness continued and the patient was soon unable to breathe or swallow. The patient became bed-bound and died four months after the onset of symptoms confirming a probable sCJD diagnosis. A post-mortem biopsy was not done to confirm a probable CJD.

## Discussion

CJD is a transmissible spongiform encephalopathy (prion disease) that occurs in the brain. Prions (PrP) are glycoproteins found in both the central and peripheral nervous system. The function of PrPC (cellular isoform) is not completely understood but is implicated in the processes of myelination and neuroprotection [6]. PrPC can be converted into PrPSC (disease-associated infectious isoform) which is the misfolded form of PrP. PrPSC is an insoluble form of the protein and leads to aggregates. Accumulation of these PRPSC aggregates causes prion diseases [6]. These aggregates cause irreversible damage to nerve cells in the brain, which quickly leads to death. The different mechanisms by which the PrPSC (misfolded) forms from PrPC (normal) classify the type of CJD.

As mentioned previously, the CDC lists three types of CJD namely sporadic, familial, and iatrogenic [4]. However, some sources list a fourth class, also including variants [7]. First, there is sporadic. This is the most common form of CJD (about 85%) [6]. This is caused by a PrPC protein sporadically misfolding. This spreads and causes further PrPC to convert to the PrPSC form. Next, there is familial CJD. This occurs when an individual inherits a prion protein gene with the mutation that will cause PrPSC to be synthesized. The third class is iatrogenic CJD. This is caused by the accidental spread through medical or surgical treatment [7]. This occurs when neurosurgical equipment is not properly washed following operations or a fluid transplant containing infected proteins. As awareness of neurodegenerative diseases has increased the occurrence of iatrogenic CJD has greatly decreased [7]. Finally, there is the CJD variant. This occurs when meat is consumed that contains bovine spongiform encephalopathy (mad cow disease) [6]. Extensive measures have been taken since the discovery of this connection to ensure that the meat that will be consumed is safe.

The prions in CJD are similar to those found in Gerstmann-Sträussler-Scheinker Disease (GSS) and Kuru. These are similar prion disorders. However, GSS is purely a genetic prion disorder. It is differentiated from CJD because it occurs earlier in life and spreads more slowly. Patients typically live about five years after diagnosis. Kuru is an almost extinct prion disorder that occurred when cannibals consumed the brains of infected individuals [8]. This was a typical practice in New Guinea until the late 1950s [8].

Clinically, CJD can be difficult to differentiate from Alzheimer's Disease. This is due to the presentation of patients with similar neurological deficits such as rapid memory loss, loss of brain function, and dementia [6]. Similar to CJD, the gold standard for diagnosing Alzheimer’s Disease requires postmortem examination [9]. However, a CSF tap examination of ⍶𝛃 plaques and tau proteins enables physicians to differentiate between CJD and Alzheimer’s Disease [9].

As mentioned, CJD is normally accompanied by severe neurological and psychological symptoms such as slurred speech, memory loss, numbness, hallucinations, ataxia, depression, anxiety, and difficulty speaking [6,10]. It is always fatal and death normally occurs within one year of diagnosis [4]. This case is unique as the patient presented with many of the typical physical symptoms of CJD but without the neurological symptoms. Patient had no memory loss, hallucinations, or typical symptoms of a degenerative neurological disorder such as CJD. As far as we are aware, this is extremely rare. We were only able to find two other cases that presented with absent mental deterioration [11,12]. In both of these cases the patients died four months later, the same time as our patient [11,12]. There are several cases that present without physical symptoms that were later identified as CJD, but not the physical symptoms without neurological symptoms [13]. The large majority of cases of sporadic CJD report significant neurological deficits. It is important that clinicians bear in mind that not all cases of CJD present the same and to not cross CJD off of your differential diagnosis when hallmark signs are not seen.

One additional finding that makes this case interesting is the indeterminate RT-QuIC test. The RT-QuIC test involves taking a CSF sample, adding normal prion proteins to the SCF sample, then incubate/shake the sample [14]. In most prion disorders, if there are abnormal proteins present, they will induce the normal prions to also become abnormal. This test has a diagnostic sensitivity of 80% and a specificity of over 90% [15]. Our patient presented with an indeterminate RT-QuIC test which is irregular for an advanced stage of CJD.

One limitation of this case is that no brain biopsy was done. Brain biopsy is the gold standard for CJD diagnosis. Without this we concluded a probable sporadic CJD. The patient met all other criteria for a definitive diagnosis of CJD and died with symptoms that have been recorded in other cases. This is interesting and shows that a patient can have CJD with an indeterminate RT-QulC test. The patient died four months following the initial presentation which led to a probable sporadic CJD diagnosis.

## Conclusions

CJD is a fatal diagnosis that typically presents with several neurological and psychological disorders. Here is presented an atypical presentation of a probable sporadic CJD with no neurological and psychological disorders except for a lagging ankle as well as an indeterminate RT-QuIC test. The patient died four months following the initial presentation of symptoms indicating a very rapid stage of progression. This concurs with the two other cases reported of patients with CJD with absent neurological symptoms. Clinicians should be aware of the normal as well as the abnormal presentations of CJD and keep it in their differential when working up patients and sudden death.
